# Determination of Seed Yield and Nutrient Element Contents of Pepper Plants Under Organic and Conventional Conditions

**DOI:** 10.3390/plants15213426

**Published:** 2026-11-04

**Authors:** Merve Şimşit, Muhammed Loay Ali, Saliha Dorak Mestan, Barış Bülent Aşık, Sevinç Başay

**Affiliations:** 1 Department of Horticulture, Faculty of Agriculture, Bursa Uludag University, Bursa 16059, Turkey; mervesimsitt@gmail.com (M.Ş.); loayali021@gmail.com (M.L.A.); 2 Department of Soil Science and Plant Nutrition, Faculty of Agriculture, Bursa Uludag University, Bursa 16059, Turkey; salihadorakmestan@uludag.edu.tr (S.D.M.); bbasik@uludag.edu.tr (B.B.A.)

**Keywords:** organic farming, *Capsicum annuum* L., fruit morphology, seed quality

## Abstract

This study evaluated the fruit morphological traits, seed yield components, physiological seed quality, and mineral element contents of seven hot pepper (*Capsicum annuum* L.) cultivars under organic and conventional production systems over two consecutive growing seasons (2022–2023). Overall, both the production system and cultivar significantly (*p* ≤ 0.05) influenced all evaluated parameters. Conventional farming yielded higher average fruit weight (19.59 g vs. 7.85 g) and provided superior physiological seed vigor, with ‘Demok’ achieving the highest germination percentage (96.00%) and ‘Tatlı Sivri Çetinel’ exhibiting the shortest mean germination time (5.02 days) and highest 1000-seed weight (5.96 g). Conversely, the organic production system promoted mean fruit length (15.13 cm vs. 12.46 cm) and generative seed set capacity. Under organic conditions, ‘Demok’ stood out by reaching the highest fruit length (18.00 cm), fruit diameter (17.86 mm), fruit weight (20.79 g), seed number per fruit (142.34 seeds/fruit), and seed weight per fruit (0.74 g/fruit). Regarding nutrient accumulation, organic management significantly enhanced fruit potassium (10,697.71 mg kg^−1^ in ‘Demok’) and iron contents, whereas conventional management resulted in higher boron and molybdenum levels. ‘Burdem 016 Demre’ accumulated the highest molybdenum (1580.41 mg kg^−1^), ‘Yalova Çorbacı Sarı Sivri’ recorded the highest iron under conventional conditions (73.58 mg kg^−1^), and ‘Sürmeli’ achieved the highest calcium (813.78 mg kg^−1^) and boron (22.02 mg kg^−1^) under organic production. In conclusion, performance under organic conditions is strictly trait- and cultivar-dependent, highlighting ‘Demok’ as a superior candidate for organic seed production when appropriate selection criteria are applied.

## 1. Introduction

Increasing environmental problems in recent years have heightened interest in production systems focused on the sustainable use of natural resources. In this context, organic farming stands out as a production system based on ecological cycles, minimizing synthetic inputs, and prioritizing environmental sustainability. Organic farming practices offer multifaceted contributions, such as preserving and enhancing soil fertility, supporting biodiversity, ensuring effective management of natural resources, and providing food security [1,2,3]. Globally, organic farming activities are currently conducted by approximately 2.8 million producers across an area exceeding 71 million hectares. In Turkey, the organic production area, which was only 1037 hectares in 1990, has grown significantly over time to exceed 626,000 hectares, covering more than 200 products [4,5]. However, the fact that Turkey’s global organic market share remains at approximately 0.1% underscores the necessity of utilizing its existing ecological potential more effectively [6].

Pepper production in Turkey holds a significant share within the vegetable production system. In 2024, total vegetable production in Turkey reached 33.6 million tons, while pepper production was recorded at approximately 3,428,038 tons during the same period. This quantity indicates that pepper accounts for approximately 10–11% of total vegetable production, highlighting the strategic importance of the crop [7]. Among vegetable species, pepper (*Capsicum annuum* L.) is one of the most economically valuable species of the Solanaceae family. Owing to its wide genetic and morphological variation, it can adapt to diverse ecological conditions [8,9]. In addition to fresh consumption, pepper is processed as pickled, paste, and dried products. Rich in ascorbic acid, capsaicin, pigments, and antioxidant compounds, it serves not only as a nutritious vegetable but also as a functional food product [10,11]. In Turkey, hot pepper types (sivri) particularly stand out in production due to their high adaptation capacity, genetic diversity, and increasing consumer demand [12,13].

Fruit load on the plant has been reported to significantly affect seed yield and quality characteristics in pepper. For example, in a study conducted under greenhouse conditions, an application with 13–15 fruits per plant yielded an average of 86 seeds per fruit, a 1000-seed weight of 7.45 g, a germination rate of 85%, and a viability rate of 86.33% [14]. In pepper cultivation, seed yield and quality parameters are substantially influenced by cultivar characteristics as well as environmental conditions and production systems. The literature indicates that parameters such as fruit length, fruit diameter, number of seeds per fruit, 1000-seed weight, and germination performance vary depending on organic and conventional production systems [15,16,17]. The readily available nutrients in conventional systems can increase seed weight, whereas greater seed formation per fruit can be observed in organic systems. However, limiting factors under organic production conditions, such as weed density and disease pressure, can negatively affect production efficiency [18,19]. Furthermore, studies have reported that seed yield and quality parameters in pepper are sensitive to nutrition and cultivation practices, which can be decisive for seed yield, germination rate, and germination index [20].

Despite the recent increase in demand for organic production and organic seeds, comparative scientific studies evaluating organic and conventional production systems, particularly in hot pepper cultivars, remain limited. This study aimed to evaluate the fruit morphological traits, fruit-based seed yield components (seeds/fruit, seed weight/fruit, 1000-seed weight), and physiological seed quality parameters (germination performance) of different hot pepper cultivars under organic and conventional farming conditions. The study is expected to contribute to demonstrating the adaptation potential of the cultivars and evaluating the advantages of organic farming systems for sustainable seed production.

## 2. Materials and Methods

### 2.1. Materials

This study was conducted during the 2022 and 2023 growing seasons in the organic and conventional experimental fields of the Faculty of Agriculture at Bursa Uludağ University, as well as in the Seed Science Laboratory of the Department of Horticulture. Seven different hot pepper (*Capsicum annuum* L.) cultivars were used as plant material: ‘Sürmeli’, ‘Tatlı Sivri Çetinel’, ‘İnce Sivri Kıl Tatlı’, ‘Burdem 016 Demre’, ‘Yalova Çorbacı Sarı Sivri’, ‘Demok’, and ‘Burkalem’. In the tables, these cultivars are designated with the codes I, II, III, IV, V, VI, and VII, respectively. The climatic data of the study site for the years 2022 and 2023 are presented in Table 1.

### 2.2. Method

The experiment was established in a Randomized Complete Block Design with three replications, containing 30 plants in each replication. With this arrangement, 21 plots were created for each production system, resulting in a total of 42 plots. Seedlings were transplanted into furrows with row spacing and intra-row spacing of 80 × 30 cm on pre-prepared plots equipped with a drip irrigation system. Seedling transplanting was carried out on 26 May 2022, in the first year, and 16 May 2023, in the second year.

For fertilization in organic production plots, only fully decomposed farmyard manure (20 tons ha^−1^) was used, while in conventional plots, 30 kg of diammonium phosphate (DAP; 18% N, 46% P_2_O_5_) was applied as a basal fertilizer. Some of the constituents of the decomposed farmyard manure used in the study, along with the characteristics of the soil in the experimental area, are presented in Table 2.

Irrigation operations were carried out 2–3 times a week using the drip irrigation method in both production systems, depending on climatic conditions and plant water requirements. Weed control was performed at regular intervals using mechanical methods.

Within the scope of measurements regarding fruit morphology, fruit length (cm) was determined with the help of a ruler, fruit diameter (mm) using a digital caliper sensitive to 0.001 mm, and fruit weight (g) with a precision balance (SELES NHB-600M, Pruiste (Anhui) Measuring Technology Co., Ltd., Bengbu, China).

In determining fruit-based seed yield components, the number of seeds per fruit (seeds/fruit), seed weight per fruit (g), and 1000-seed weight (g) were calculated according to ISTA [22]. rules, whereas physiological seed quality parameters (germination percentage, germination index, and mean germination time) were evaluated through laboratory germination tests.

Thousand-Seed Weight Equation:

TSW = (Total weight of sown seeds (g)/Counted number of seeds) × 1000

Germination tests were carried out in four replications for each cultivar, with 50 seeds used in each replication. The tests were conducted in accordance with ISTA rules [22]. Seeds were placed on germination paper previously moistened with distilled water in Petri dishes without touching each other and incubated in a germination cabinet (Nüve ES 120 and Nüve FN 500P, Genamed (GenaMed), Bursa, Turkey) set at a temperature of 25 °C, The Petri dishes were wrapped with stretch film to prevent moisture loss, Germination counts were performed daily for 14 days starting from the third day, based on seeds showing a radicle emergence of at least 2 mm, Germination percentage (%), germination index according to Kaya [23], and mean germination time were calculated according to the method of Ellis and Roberts [24].

Germination Index Equation:

GI = ∑ (n/D)

where GI represents the speed of germination, n is the number of normal seedlings germinated on each day, and D is the day of germination.

Mean Germination Time Equation:

MGT = ∑ (n × D)/∑ n

where MGT is the mean germination time, n is the number of seeds germinated on day D, and D is the number of days elapsed from initiation.

Seed moisture content was determined according to ISTA rules [22] using the low constant temperature oven method. Seed samples were dried in an oven (Nüve FN 500P) at 103 °C for 17 h. Following the drying process, the seeds were kept in a desiccator for 30 min, after which their final weights were measured.

Seed Moisture Content Equation:

Moisture Content (%) = [(ISW − FSW)/ISW] × 100

where ISW is Initial Seed Weight (g), and FSW is Final Seed Weight (g).

The dried seed samples were used for macro and micro plant nutrient elements content analysis. The results were calculated on a dry matter basis. Seed samples were ground and prepared for the analysis of macro- and micronutrient content. A 0.25 g portion of each sample was weighed and subjected to wet digestion using a Berghof MWS-2 microwave digestion system with 3 mL of HNO_3_ and 4 mL of H_2_O_2_ at 180 °C for 30 min. Following digestion, the sample solutions were diluted to a final volume of 50 mL using deionized water containing 2% nitric acid. Nitrogen (N) content was determined via the Kjeldahl method using a Büchi K-437/K-350 total digestion/distillation unit. Total elemental content—including phosphorus (P), potassium (K), calcium (Ca), sodium (Na), iron (Fe), manganese (Mn), copper (Cu), and zinc (Zn)—was subsequently analyzed using inductively coupled plasma optical emission spectrometry (ICP-OES; Perkin Elmer Optima 2100 DV) [25]. For the elemental analyses, five-point calibration curves were generated using 1000 ppm ICP standard solutions (Merck KGaA, Darmstadt, Germany).

Three independent weighing and digestion procedures were performed for each sample. To monitor potential contamination from reagents, reagent blanks were also included in the analyses. The results are expressed on a dry matter basis. [25].

### 2.3. Statistical Analysis

Data obtained in this study were analyzed using IBM SPSS Statistics for Windows, Version 28.0 (IBM Corp., Armonk, NY, USA). The experimental layout followed a Randomized Complete Block Design (RCBD) with three replications per system. To determine the main effects of growing system (2 levels: Organic and Conventional), cultivar (7 levels), and year (2 levels: 2022 and 2023), a three-way Analysis of Variance (ANOVA) based on a 3-factor factorial arrangement was performed by partitioning total variation into between-group and within-group components to statistically evaluate differences among means [26]. In this model, blocks were included as random effects to account for spatial variability. In addition to the main effects of growing system, cultivar, and year, two-way (System X Cultivar, System X Year, Cultivar X Year) and three-way (System X Cultivar X Year) interactions among factors were evaluated. Assessing interaction effects in factorial designs is crucial, as it indicates whether the effect of one factor on the evaluated traits depends on the levels of other factors [27]. When ANOVA indicated statistically significant differences, Duncan’s multiple range test was applied to compare mean values and separate them into statistical groups [28]. The significance level was set at 5% for all statistical analyses, and results were evaluated at *p* ≤ 0.05.

## 3. Results and Discussion

In both conventional and organic production systems, the differences among cultivars in terms of fruit length, fruit diameter, and average fruit weight were found to be statistically significant at *p* ≤ 0.05 (Table 3).

In terms of fruit length, higher values were obtained under organic production (16.29 cm) compared with conventional production (14.29 cm). With respect to the trial years, the second year (15.79 cm) showed a higher value than the first year (14.78 cm). For fruit diameter and mean fruit weight, higher values were also obtained under organic production; however, the difference between production systems was not statistically significant for fruit diameter (*p* = 0.1596), whereas it was significant for mean fruit weight (*p* = 0.0028). Differences between years were not statistically significant for either trait (*p* = 0.9487 and *p* = 0.9516, respectively) (Table 3).

In terms of fruit length, the organic production system yielded better results than the conventional production system in the ‘Demok’ and ‘İnce Sivri Kıl Tatlı’ cultivars, with values of 18.00 cm and 17.85 cm, respectively (Table 4). Regarding fruit diameter and fruit weight, the ‘Demok’ cultivar exhibited the highest values under organic production, at 17.86 mm and 20.79 g, respectively.

In a study conducted in North Macedonia comparing *Capsicum annuum* L. genotypes under organic and conventional production systems in terms of fruit traits, fruit diameter was measured as 5.2 cm, mean fruit weight as 32.4 g, and fruit length as 12.8 cm in organic production, whereas these values were determined as 6.1 cm, 45.7 g, and 11.6 cm, respectively, in conventional production [29]. These results show parallelism with our study regarding fruit length and fruit diameter.

In a study evaluating five different pepper cultivars, fruit weight ranged from 112.80 g to 217.99 g, with the highest fruit length determined as 16.2 cm in the ‘Red Bell’ cultivar [30]. While these results partially align with our findings in terms of fruit length, they show differences regarding fruit weight.

In a study investigating the morphological traits affecting fruit weight in local *Capsicum annuum* var. *grossum* pepper cultivars collected from Romania, it was reported that 52% of the variability in fruit weight was attributed to four morphological traits, particularly fruit length and fruit diameter. The average fruit length, fruit diameter, and mean fruit weight of the cultivars were determined as 7.2 cm, 2.4 cm, and 85.3 g, respectively [31]. These results are consistent with our study regarding the relationship between fruit weight, fruit length, and diameter.

In the study by Luitel et al. [32], 55 different pepper (*Capsicum annuum* L.) genotypes were evaluated for fruit characteristics; fruit length ranged between 4.0 and 16.2 cm, fruit diameter between 4.8 and 10.7 mm, and mean fruit weight varied between 27.3 and 200 g depending on the genotypes, Additionally, the fruit length-to-width ratio varied from 0.6 to 3.0, and pericarp thickness ranged from 1.8 to 10.7 mm.

Fruit morphology is influenced by genetic and developmental processes, including cell division, cell expansion, and hormonal regulation [33]. These processes provide a general biological framework for understanding variation in fruit length, diameter, and weight among cultivars. However, the present study evaluated fruit morphological traits rather than the underlying molecular or hormonal mechanisms. Therefore, the differences observed among cultivars and production systems should be interpreted on the basis of the measured traits, while the mechanisms described in the literature serve as potential explanations rather than processes demonstrated by the present experiment.

In terms of the number of seeds per fruit, the organic production system in the ‘Demok’ cultivar yielded better results than the conventional production with 142.34 seeds/fruit (Table 5). Regarding 1000-seed weight, the ‘Tatlı Sivri Çetinel’ cultivar exhibited the highest value in conventional production at 5.96 g. Similarly, Demirkaya and Demirkaya and Güneş [20] reported that different effective microorganisms and nitrogen doses had distinct effects on seed yield and germination index in pepper. Zhigila et al. [34], in their study on five different *Capsicum annuum* varieties (var. *abbreviatum*, var. *annuum*, var. *accuminatum*, var. *grossum*, and var. *glabriusculum*), reported that the number of seeds per fruit ranged from 41.3 to 108.4 seeds. In our study, the number of seeds per fruit ranged from 70.21 to 142.34 seeds. The differences between the studies are thought to stem from the variation in *Capsicum annuum* genotypes (cultivars/varieties) utilized in the research.

When evaluating the number of seeds per fruit and 1000-seed weight together, production systems appear to exert contrasting effects on generative seed setting capacity (seed number per fruit) and individual seed filling (1000-seed weight) rather than cumulative field seed yield. Under organic production conditions, the ‘Demok’ cultivar achieved the highest number of seeds per fruit with 142.34 seeds/fruit, whereas under conventional production conditions, the ‘Tatlı Sivri Çetinel’ cultivar stood out with a 1000-seed weight of 5.96 g. This indicates that certain cultivars can produce a higher number of seeds per fruit under organic conditions, whereas conventional production may enhance seed fullness and unit seed weight. In particular, the high seed count observed in the ‘Demok’ cultivar under organic production suggests a robust generative adaptation under organic conditions. Conversely, the high 1000-seed weight attained by ‘Tatlı Sivri Çetinel’ in conventional production indicates that readily available nutrients may exert a positive effect on seed filling.

In a study conducted by Dilrukshi et al. [14] on *Capsicum annuum* Grossum group cultivars, an average of 86 seeds per fruit and a 1000-seed weight of 7.45 g were reported. In another study on the Huacle pepper native to Oaxaca, Mexico, the 1000-seed weight was determined as 5.32 g, the mean moisture content as 7.2%, and the number of seeds per fruit as 110 seeds/fruit [30].

Ayala-Villegas et al. [35], in their investigation of the physical and physiological quality of pepper (*Capsicum annuum*) seeds depending on fruit development stages, determined the seed moisture content as 7.6%, the highest 1000-seed weight as 7.45 g, and the average number of seeds per fruit as 86 seeds/fruit.

Tetteh et al. [36] evaluated two different *Capsicum annuum* L. cultivars, reporting 100-seed weights for the ‘Legon 18’ cultivar at the initial, semi-ripe, and fully ripe stages as 0.473 g, 0.499 g, and 0.503 g, respectively; for the ‘BAG 14/001’ cultivar, these values were recorded as 0.323 g, 0.415 g, and 0.443 g, respectively, In both cultivars, the highest 100-seed weight was obtained at the fully ripe stage, These findings are consistent with our study regarding the effect of harvest maturity on seed weight and physiological quality in pepper.

Regarding seed weight per fruit, the organic production system in the ‘Demok’ cultivar yielded better results than conventional production, reaching 0.74 g. In terms of moisture content, the ‘Burdem 016 Demre’ cultivar exhibited the highest value under organic production at 8.60% (Table 6).

In a study conducted by Arslan et al. [37] on different pepper (*Capsicum annuum* L.) genotypes, seed weight per fruit was reported to range between 0.30 and 0.72 g. Chakrabarty [38] reported that seed weight per fruit in various pepper genotypes varied between 0.15 and 0.53 g, with a mean of 0.30 g. Furthermore, Kaya and Demir [39] observed that seed weight per fruit in eggplant (*Solanum melongena* L.) ranged from 0.25 to 0.64 g.

In a study by Başay [40], investigating seed yield and quality under organic and conventional production conditions in *Capsicum annuum* L. ‘Kandil Dolma’, mean seed weight per fruit was recorded as 1.7 g in organic production and 3.25 g in conventional production. Across harvest periods, mean seed weight per fruit under organic production was 2.1 g in the first harvest and 1.5 g in the second harvest; under conventional production, these values were 4.2 g and 2.7 g, respectively.

In the study by Martínez-Sánchez et al. [41] evaluating the organic production of Huacle pepper native to Oaxaca, Mexico, the number of seeds per fruit was determined as 110 seeds/fruit, 1000-seed weight as 5.32 g, and seed moisture content as 7.2%. These results align with our study in terms of number of seeds per fruit, 1000-seed weight, and seed moisture content.

In an investigation by Ayala-Villegas et al. [35] on the physical and physiological quality of *Capsicum annuum* seeds harvested at different maturity stages, seed moisture content was recorded as 7.6% and 1000-seed weight as 6.5 g, with peak seed quality achieved at full maturity. These findings parallel our results regarding seed moisture content and germination quality, while displaying slight variations in 1000-seed weight.

Regarding physiological seed quality traits (germination percentage, germination index, and mean germination time), the conventional production system yielded higher results in the ‘Demok’ cultivar at 96.00% compared to organic production. For mean germination index, the conventional production system in the ‘Tatlı Sivri Çetinel’ cultivar performed better than organic production, reaching 9.60. In line with this, the shortest mean germination time was observed in the ‘Tatlı Sivri Çetinel’ cultivar under conventional production at 5.02 days. In organic production, the shortest mean germination time was recorded in the ‘Burdem 016 Demre’ cultivar at 5.73 days. Conversely, the ‘Yalova Çorbacı Sarı Sivri’ cultivar under organic production displayed the longest mean germination time at 6.72 days, demonstrating lower performance in terms of germination speed. Overall, the conventional production system provided more favorable conditions for enhancing physiological seed vigor, whereas organic conditions primarily influenced generative seed set capacity (Table 7). When evaluating mean germination time, lower values denote faster germination. Indeed, Duman [15] noted that seeds from the first harvest reached mean germination times of 4.3–4.9 days compared to 5.2–5.6 days for the second harvest, indicating higher germination speed in early-harvested seeds. In line with this, the shortest mean germination time was observed in the ‘Tatlı Sivri Çetinel’ cultivar under conventional production at 5.02 days. In organic production, the shortest mean germination time was recorded in the ‘Burdem 016 Demre’ cultivar at 5.73 days. Conversely, the ‘Yalova Çorbacı Sarı Sivri’ cultivar under organic production displayed the longest mean germination time at 6.72 days, demonstrating lower performance in terms of germination speed.

In a study conducted by Yıldırım and Demir [42] on various *Capsicum annuum* L. cultivars grown under organic and conventional conditions, the mean germination time ranged from 4.2 to 5.8 days in organic production and from 4.3 to 6.0 days in conventional production.

In a study on pepper seeds, it was reported that seaweed extract priming in the ‘Demre Sivri’ pepper cultivar reduced the mean germination time from 7.2 days to 5.6 days and increased the germination rate to 94.5% [18].

Nogueira et al. [43], in their study on the ‘Magda’ pepper cultivar (*Capsicum annuum* L.), found that seeds harvested at the green maturity stage (51 days after anthesis) under organic conditions exhibited a germination rate above 90%.

While Mahmud et al. [44] reported germination rates ranging between 36.67% and 68.0% in sweet pepper seeds, Dzib-Ek et al. [17] determined germination rates between 82% and 86% in *Capsicum annuum* L. var. *glabriusculum*. In our study, the highest mean germination rate was recorded as 96.00% under conventional production conditions, which was higher than the germination rates reported in both studies. This difference can be attributed to variations in the genotypes used, cultivation conditions, and the physiological quality of the seeds,

Differences in macroelement contents of the evaluated cultivars were analyzed across both conventional and organic production systems, and the production system was found to exert a statistically significant effect (*p* ≤ 0.05), particularly on K content (Table 8).

Fruit K content was higher in the organic production system (8605.24 mg kg^−1^) compared to conventional production (8162.29 mg kg^−1^). In contrast, no significant differences were observed between production systems regarding N, P, Ca, Mg, and Na contents. When evaluated across trial years, no statistically significant differences were detected for any of the analyzed macroelement contents (Table 7). In the second year, N (2.60%), P (5687.22 mg kg^−1^), and K (8550.33 mg kg^−1^) contents were higher than in the first year, Conversely, the lowest N (2.53%), P (5591.11 mg kg^−1^), and K (8211.79 mg kg^−1^) values were recorded in the first year, Calcium content was higher in the first year at 670.79 mg kg^−1^ and decreased to 623.64 mg kg^−1^ in the second year.

Although N, P, and K contents were numerically higher in the second year than in the first year, the differences between years were not statistically significant (Table 9). Previous studies have reported that environmental factors, such as temperature, light intensity, relative humidity, and irrigation frequency, may influence nutrient uptake in plants [45]. Taiz et al. [46] also indicated that variations in temperature and light intensity, particularly under greenhouse conditions, may affect nutrient uptake through changes in photosynthesis and root–shoot balance. Furthermore, the accumulation of organic matter and changes in soil microbial activity may influence N and P mineralization and, consequently, nutrient concentrations in plant tissues [47,48]. Studies on *Capsicum annuum* L. have also reported variations in fruit mineral composition among growing years, even within the same cultivars [49,50]. These findings provide possible explanations for the numerical variations observed in the present study. However, these factors were not directly measured in the present study; therefore, their contributions to the observed numerical variations cannot be established.

Each nutrient plays a specific role in the physiological processes of plant growth. An insufficient supply of a nutrient is a primary cause of poor growth and reduced yield. The same principle applies to the nutrient content of seeds. Studies have shown a strong correlation between nutrient management and the number of seeds per plant, as well as seed yield and quality [51,52,53].

Nitrogen and phosphorus play the most important role in seed production and development. The work concerning the effects of nitrogen on seed production and its relationship with seed protein has mainly been done. Phosphorus (P) is found in nucleic acids, nucleotides, and phospholipids and also plays a vital role in plant metabolism. This element is important for root development and growth, particularly in the early stages of plant life. Phosphorus has therefore always been considered an essential element for the formation of the inherent parts of cells, and as seeds consist of cells as units, P must be necessary for the formation of new seeds. Potassium (K) is the most abundant cation in plants; it acts as an osmotic regulator. It is essential for the synthesis of chlorophyll, proteins, carbohydrates, and fats, and is an important contributor to enzyme activity; therefore, it is an essential element in plants and seed development [48].

In the inter-annual evaluation, the increase in N, P, and K contents observed in the second year can be attributed to the fact that plant nutrient uptake is significantly influenced by environmental factors such as temperature, light intensity, relative humidity, and irrigation frequency [45], Similarly, Taiz et al. [46] reported that temperature and light variations between years, particularly under greenhouse conditions, can alter nutrient uptake by affecting the photosynthetic rate and consequently the root–shoot balance, Drinkwater et al. [47] and Marschner [48] stated that the accumulation of organic matter and increased microbial activity in the soil over time can accelerate N and P mineralization, leading to higher nutrient concentrations in plant tissues, Briefly, it can be clearly stated that changes in environmental conditions exert a direct effect on plant nutrient uptake, Indeed, studies conducted on *Capsicum annuum* L. have reported that significant variations in fruit mineral composition can occur even within the same cultivars depending on the year [49,50]. These results are in full agreement with the findings obtained in our study.

When evaluated on a cultivar basis, in the conventional production system, the highest N content was determined in ‘Tatlı Sivri Çetinel’ at 2.82%, while the lowest values were recorded in ‘İnce Sivri Kıl Tatlı’ and ‘Burkalem’ at 2.43%, In terms of P content, ‘Demok’ ranked in the top group with 6192.96 mg kg^−1^, whereas the lowest value was observed in ‘İnce Sivri Kıl Tatlı’ at 4942.56 mg kg^−1^, Regarding potassium (K) content, ‘Tatlı Sivri Çetinel’ exhibited the highest value with 8855.50 mg kg^−1^, while ‘İnce Sivri Kıl Tatlı’ and ‘Burdem 016 Demre’ were in the lowest group, For Ca, ‘Burdem 016 Demre’ provided the highest value at 759.38 mg kg^−1^, and for Mg content, ‘Demok’ ranked first with 3143.81 mg kg^−1^, The Na content ranged between 257.32 and 346.55 mg kg^−1^ among cultivars, although the differences were not statistically significant (Table 9).

In the organic production system, statistically significant differences (*p* < 0.05) were determined among cultivars in terms of potassium content, The highest K content was determined in ‘Demok’ at 10,697.71 mg kg^−1^, and the lowest value was recorded in ‘İnce Sivri Kıl Tatlı’ at 7665.94 mg kg^−1^, Although no statistically significant differences were found among cultivars for other macroelement contents, nitrogen content ranged from 2.46% to 2.75%, phosphorus content from 5138.12 mg kg^−1^ in ‘İnce Sivri Kıl Tatlı’ to 6464.11 mg kg^−1^ in ‘Demok’. Ca content ranged from 596.82 mg kg^−1^ in ‘Demok’ to 813.78 mg kg^−1^ in ‘Sürmeli’, while Mg content varied from 2739.13 mg kg^−1^ in ‘İnce Sivri Kıl Tatlı’ to 3136.37 mg kg^−1^ in ‘Demok’. Na content ranged from 236.69 mg kg^−1^ in ‘Demok’ to 339.50 mg kg^−1^ in ‘Sürmeli’ (Table 9).

The observed differences in macroelement contents among pepper cultivars indicate variation in their mineral composition under the evaluated production conditions [48,54]. Previous studies have suggested that genetic differences, root characteristics, root exudates, and mycorrhizal associations may influence nutrient uptake, particularly phosphorus acquisition [55,56]. Potassium plays important roles in osmotic regulation, stomatal movement, and enzyme activation and may therefore be associated with differences in plant growth and fruit quality [50,57]. In addition, the availability and uptake of mineral elements may be influenced by interactions among nutrients, including competition between Na, K, Ca, and Mg [57,58]. Soil biological processes, including the decomposition of organic materials and microbial activity, may also affect nutrient availability to plants [48,59]. However, root characteristics, mycorrhizal associations, soil microbial activity, fertilizer composition, and nutrient availability in the root zone were not directly assessed in the present study. Therefore, these processes should be considered possible explanations rather than confirmed mechanisms underlying the observed differences. Furthermore, because different fertilizer inputs were used in the organic and conventional production systems, the observed differences should not be attributed exclusively to the production systems themselves.

When the microelement contents of hot pepper cultivars grown in conventional and organic production systems were evaluated, the production system was found to exert a statistically significant effect (*p* ≤ 0.05) on Fe, B, and Mo contents (Table 10).

In the organic production system, Fe content was higher (66.40 mg kg^−1^) compared to conventional production (60.57 mg kg^−1^), whereas boron (B) and molybdenum (Mo) contents were higher in conventional production at 16.04 mg kg^−1^ and 974.20 mg kg^−1^, respectively, than in organic production (11.61 mg kg^−1^ and 389.13 mg kg^−1^), Conversely, no statistically significant differences were detected between production systems regarding Cu, Zn, and Mn contents.

When the results were compared between the two growing years, a statistically significant difference (*p* ≤ 0.05) was observed only in Fe content, which was higher in the second year (65.53 mg kg^−1^) than in the first year (61.41 mg kg^−1^). No statistically significant differences were detected between years for Cu, Zn, Mn, B, or Mo contents. Previous studies have reported that organic acids may increase Fe solubility and influence its availability for plant uptake (Marschner [48]; Alloway [60]). Other studies have also suggested that organic fertilizer applications may affect Fe availability [61,62]. However, soil organic matter, microbial activity, organic acid concentrations, and Fe availability in the root zone were not directly measured in the present study. Therefore, these factors may be considered possible explanations for the higher Fe content observed in the second year, but their specific contributions cannot be established from the present results.

Differences in the microelement contents of the evaluated cultivars were analyzed across both conventional and organic production systems, and the production system was found to exert a statistically significant effect (*p* ≤ 0,05), particularly on Fe, B, and Mo contents (Table 11).

Among the hot pepper cultivars grown under conventional production conditions, the highest Fe content was determined in ‘Yalova Çorbacı Sarı Sivri’ (73.58 mg kg^−1^), followed by ‘Burkalem’ (70.03 mg kg^−1^) and ‘Tatlı Sivri Çetinel’ (69.05 mg kg^−1^), The lowest Fe content was recorded in ‘Burdem 016 Demre’ (47.45 mg kg^−1^), Regarding Zn content, ‘Tatlı Sivri Çetinel’ exhibited the highest value (36.00 mg kg^−1^), while ‘Demok’ (23.54 mg kg^−1^) had the lowest Zn content, In terms of Mn content, ‘Burkalem’ recorded the highest value (30,88 mg kg^−1^), whereas ‘Demok’ (22.54 mg kg^−1^) remained at the lowest level, For boron content, ‘İnce Sivri Kıl Tatlı’ (22.43 mg kg^−1^) and ‘Yalova Çorbacı Sarı Sivri’ (20.61 mg kg^−1^) stood out, In terms of molybdenum, the ‘Burdem 016 Demre’ cultivar showed the highest value (1580.41 mg kg^−1^).

The observed differences in microelement contents among pepper cultivars indicate variation in their mineral composition under the evaluated production conditions (Table 10). Previous studies have suggested that cultivar-related differences in root function and Fe-chelate formation may influence Fe uptake [49]. Genetic variation may also contribute to differences in Zn uptake, transport, and accumulation among cultivars [62,63]. Although boron uptake may be influenced by interactions between plant genotype and environmental conditions [64], this interaction was not specifically tested in the present study. In addition, soil pH and redox conditions may affect Mo availability and uptake [61]. However, root function, Fe-chelate formation, Zn transport mechanisms, soil pH, and redox conditions were not directly assessed in the present study. Therefore, these factors should be considered potential explanations based on previous research rather than confirmed mechanisms responsible for the observed differences among cultivars.

## 4. Conclusions

This study demonstrates that hot pepper (*Capsicum annuum* L.) performance under organic and conventional farming conditions cannot be generalized, as it is strictly dependent on both the specific trait measured and the production system. Conventional farming generally provided superior conditions for individual seed filling and physiological seed vigor, as evidenced by higher 1000-seed weight, germination percentage, and faster germination speed. In contrast, the organic production system promoted fruit length and generative seed-setting capacity (seeds per fruit and seed weight per fruit), while also enhancing fruit potassium and iron accumulation.

Furthermore, cultivars exhibited distinct genotype × production system interactions. For instance, ‘Tatlı Sivri Çetinel’ stood out in conventional production regarding 1000-seed weight and germination index, whereas ‘Demok’ showed exceptional generative adaptation under organic conditions by achieving the highest fruit morphological dimensions and fruit-based seed yield components. In conclusion, there is no single production system or cultivar that excels across all performance criteria. Sustainable and successful organic hot pepper seed production requires selecting cultivars adapted to specific trait targets—such as generative seed yield vs. seed vigor—rather than relying on a standardized management approach. Each nutrient plays a specific role in the physiological processes of plant growth. An insufficient supply of a nutrient is a primary issue that leads to poor growth and reduced yield. The same principle applies to the nutrient content of seeds. Therefore, in addition to the parameters mentioned above, the amount of nutrients contained in the seed should also be considered. These findings provide a scientific framework for targeted cultivar selection in organic seed production, while future research should explore multi-location trials and organic fertilization regimes to further refine cultivar-specific recommendations.

## Figures and Tables

**Table 1:** Monthly climatic data for the Nilüfer/Bursa region during the study period (2022–2023) [21].

Months	Period	Monthly Max Temp (°C)	Monthly Avg Temp (°C)	Monthly Avg Rel Humidity (%)	Total Precip (kg/m^2^)
March	2022	23.6	4.8	75.9	60.6
	2023	20.8	9.7	78.4	62.4
April	2022	29.4	14.0	68.0	27.7
	2023	25.5	12.8	77.8	99.8
May	2022	35.8	17.8	65.1	16.4
	2023	33.4	16.9	77.7	42.8
June	2022	32.8	22.3	71.3	129.2
	2023	33.3	24.3	63.0	0.0
July	2022	34.6	24.0	61.5	2.1
	2023	42.8	26.1	57.8	0.0
August	2022	34.7	25.3	69.6	40.3
	2023	39.4	27.2	60.2	0.0
September	2022	36.9	20.7	65.2	39.2
	2023	39.4	22.6	62.9	0.0
October	2022	25.2	15.1	73.5	85.6
	2023	27.8	16.9	68.2	42.3
November	2022	18.7	10.4	80.1	110.5
	2023	20.5	12.0	75.6	78.4

**Table 2:** Some characteristics of the decomposed farmyard manure and the experimental field soil used in the study.

Properties	FYM	Soil	Class
pH	7.39	7.56	Slightly alkaline
EC, µS cm^−1^	2355	368	Nonsaline
Organic matter	59.5% ^a^	1.57	Low
Total N	1.33% ^a^	0.152 ^a^	Modarete
C/N ratio	26,03	-	
Phosphorus, mg kg^−1^	0.47% ^a^	7.31 ^b^	Low
Potassium, mg kg^−1^	1.05% ^a^	213.5 ^c^	Sufficient
Calcium, mg kg^−1^	0.74% ^a^	1544.0 ^c^	Sufficient
Magnesium, mg kg^−1^	0.33% ^a^	75.14 ^c^	Low
İron, mg kg^−1^	795.23 ^b^	12.3 ^b^	Medium
Copper, mg kg^−1^	78.52 ^b^	1.25 ^b^	Medşum
Zinc, mg kg^−1^	50.40 ^b^	1.32 ^b^	low
Manganese, mg kg^−1^	150.3 ^b^	25.89 ^b^	Medium

^a^: total, ^b^: available, ^c^: exchangeable, FYM: decomposed farmyard manure.

**Table 3:** Fruit length (cm), fruit diameter (mm), and average fruit weight (g) measurements of hot pepper cultivars in conventional and organic production systems during the 1st and 2nd years.

Cultivation System/Year	Fruit Length (cm)	Fruit Diameter (mm)	Avg Fruit Weight (g)
Conventional	14.29 b	12.54	9.70 b
Organic	16.29 a	13.13	11.82 a
Year I	14.78	12.85	10.74
Year II	15.79	12.83	10.78

Letters indicate different groups at the *p* ≤ 0.05 level.

**Table 4:** Fruit morphological characteristics of hot pepper cultivars under organic and conventional production systems.

Cultivars	Fruit Length (cm)		Fruit Diameter (mm)		Average Fruit Weight (g)	
	Conventional	Organic	Conventional	Organic	Conventional	Organic
I	12.85 d	15.12 bc	12.30 b	12.43 c	13.70 a	6.77 d
II	11.13 e	13.57 c	14.08 a	12.41 c	8.70 cd	9.40 c
III	16.02 a	17.85 a	10.58 c	8.98 e	9.69 bcd	8.83 c
IV	12.68 d	15.57 b	14.83 a	14.90 b	12.32 abc	14.74 b
V	15.40 ab	14.32 bc	13.83 a	12.44 c	11.87 abcd	8.59 c
VI	13.99 c	18.00 a	13.88 a	17.86 a	13.28 ab	20.79 a
VII	14.83 bc	14.06 bc	10.24 c	10.79 d	8.17 d	8.82 c

Letters indicate different groups at the *p* ≤ 0.05 level. I: Sürmeli, II: Tatlı Sivri Çetinel, III: İnce Sivri Kıl Tatlı, IV: Burdem 016 Demre, V: Yalova Çorbacı Sarı Sivri, VI: Demok, VII: Burkalem.

**Table 5:** Seed yield components (number of seeds per fruit and 1000-seed weight) of hot pepper cultivars under organic and conventional production systems.

Cultivars	Number of Seeds per Fruit (Seeds/Fruit)		Thousand Seed Weight (g)	
	Conventional	Organic	Conventional	Organic
I	82.38 d	110.80 bc	5.77 ab	5.26 a
II	93.31 c	98.38 cd	5.96 a	5.04 abc
III	79.54 d	76.40 e	5.00 bc	4.68 bcd
IV	105.88 b	110.83 bc	5.36 abc	5.25 a
V	133.91 a	117.78 b	4.75 c	4.42 d
VI	101.42 b	142.34 a	4.56 c	5.19 ab
VII	70.21 e	86.56 de	4.70 c	4.53 cd

Letters indicate different groups at the *p* ≤ 0,05 level. I: Sürmeli, II: Tatlı Sivri Çetinel, III: İnce Sivri Kıl Tatlı, IV: Burdem 016 Demre, V: Yalova Çorbacı Sarı Sivri, VI: Demok, VII: Burkalem.

**Table 6:** Reproductive yield components (seed weight per fruit) and seed moisture content of hot pepper cultivars under organic and conventional production systems.

Cultivars	Seed Weight per Fruit (g/Fruit)		Moisture Content (%)	
	Conventional	Organic	Conventional	Organic
I	0.44 cd	0.57 bc	7.67 ab	8.10 b
II	0.52 bc	0.46 cd	7.42 ab	7.85 bc
III	0.35 de	0.34 d	6.67 c	7.85 bc
IV	0.66 a	0.60 b	7.42 ab	8.60 a
V	0.64 ab	0.53 bc	7.92 a	8.10 b
VI	0.44 cd	0.74 a	7.17 bc	7.85 bc
VII	0.29 e	0.38 d	7.17 bc	7.60 c

Letters indicate different groups at the *p* ≤ 0.05 level. I: Sürmeli, II: Tatlı Sivri Çetinel, III: İnce Sivri Kıl Tatlı, IV: Burdem 016 Demre, V: Yalova Çorbacı Sarı Sivri, VI: Demok, VII: Burkalem.

**Table 7:** Physiological seed quality parameters (germination percentage, mean germination index, and mean germination time) of hot pepper cultivars under organic and conventional production systems.

Cultivars	Germination Percentage (%)		Mean Germination Index		Mean Germination Time (Days)	
	Conventional	Organic	Conventional	Organic	Conventional	Organic
I	88.25 e	74.75 e	7.60 f	7.10 c	6.40 a	5.83 f
II	92.75 b	69.00	9.60 a	6.17 f	5.02 f	6.06 d
III	91.00 d	88.25 b	8.18 e	7.72 b	5.89 c	6.31 c
IV	91.75 cd	83.66 c	8.33 d	7.73 b	5.79 d	5.73 g
V	87.25 f	81.50 d	7.26 g	6.93 d	6.36 b	6.72 a
VI	96.00 a	75.75 e	8.92 c	6.66 e	5.91 c	6.57 b
VII	92.00 bc	93.00 a	9.26 b	7.98 a	5.32 e	5.93 e

Letters indicate different groups at the *p* ≤ 0.05 level. I: Sürmeli, II: Tatlı Sivri Çetinel, III: İnce Sivri Kıl Tatlı, IV: Burdem 016 Demre, V: Yalova Çorbacı Sarı Sivri, VI: Demok, VII: Burkalem.

**Table 8:** Macroelement (N, P, K, Ca, Mg, Na) contents of hot pepper cultivars in conventional and organic production systems during the 1st and 2nd years.

Cultivation System	N (%)	P (mg kg^−1^)	K (mg kg^−1^)	Ca (mg kg^−1^)	Mg (mg kg^−1^)	Na (mg kg^−1^)
Conventional	2.57	5523.07	8162.29 b	629.92	2884.65	293.85
Organic	2.57	5758.13	8605.24 a	664.94	2925.52	274.19
Year						
I.	2.53	5591.11	8211.79	670.79	2880.15	283.57
II.	2.60	5687.22	8550.33	623.64	2930.13	284.72

Letters indicate different groups at the *p* ≤ 0.05 level. I: Sürmeli, II: Tatlı Sivri Çetinel.

**Table 9:** Macroelement (N, P, K, Ca, Mg, Na) contents of hot pepper cultivars under conventional and organic production systems.

Cultivars		N (%)	P (mg kg^−1^)	K (mg kg^−1^)	Ca (mg kg^−1^)	Mg (mg kg^−1^)	Na (mg kg^−1^)
**Conventional**	I	2.51 bc	5188.77 cd	7760.64 de	513.45 d	2753.45 cd	257.74
	II	2.82 a	5681.86 b	8855.50 a	721.82 ab	2839.74 bc	304.55
	III	2.43 b	4942.56 d	7631.51 e	648.85 abc	2613.02 d	282.13
	IV	2.67 ab	5515.87 bc	7566.73 e	759.38 a	2913.41 bc	257.32
	V	2.65 b	5417.12 bc	8098.68 cd	557.50 cd	2936.14 abc	336.95
	VI	2.48 c	6192.96 a	8824.44 ab	617.74 bcd	3143.81 a	346.55
	VII	2.43 b	5722.44 b	8398.54 bc	590.73 cd	2993.00 ab	271.71
**Organic**	I	2.69	5809.40	8660.34 bc	813.78	3059.82	339.50
	II	2.55	5138.12	9131.75 b	658.30	2873.65	253.56
	III	2.46	5138.12	7665.94 c	614.24	2739.13	291.07
	IV	2.75	5648.42	8235.97 bc	631.58	2895.27	258.62
	V	2.53	5789.01	8025.43 bc	762.54	2834.52	293.63
	VI	2.52	6464.11	10697.71 a	596.82	3136.37	236.69
	VII	2.47	5527.83	7756.68 bc	620.32	2903.40	256.65

Letters indicate different groups at the *p* ≤ 0.05 level. I: Sürmeli, II: Tatlı Sivri Çetinel, III: İnce Sivri Kıl Tatlı, IV: Burdem 016 Demre, V: Yalova Çorbacı Sarı Sivri, VI: Demok, VII: Burkalem.

**Table 10:** Microelement (Fe, Cu, Zn, Mn, B, and Mo) contents of hot pepper cultivars in conventional and organic production systems during the 1st and 2nd years.

Cultivation System	Fe (mg kg^−1^)	Cu (mg kg^−1^)	Zn (mg kg^−1^)	Mn (mg kg^−1^)	B (mg kg^−1^)	Mo (mg kg^−1^)
Conventional	60.57 b	12.65	28.75	27.31	16.04 a	974.20 a
Organic	66.40 a	13.01	30.02	27.03	11.61 b	389.13 b
Year						
I.	61.41b	12.60	29.64	27.29	13.001	666.20
II.	65.53a	13.07	29.11	27.05	14.66	704.64

Letters indicate different groups at the *p* ≤ 0.05 level. I: Sürmeli, II: Tatlı Sivri Çetinel.

**Table 11:** Microelement (Fe, Cu, Zn, Mn, B, and Mo) contents of hot pepper cultivars under conventional and organic production systems.

Cultivars		Fe (mg kg^−1^)	Cu (mg kg^−1^)	Zn (mg kg^−1^)	Mn (mg kg^−1^)	B (mg kg^−1^)	Mo (mg kg^−1^)
**Conventional**	I	56.52 c	13.06	26.33 c	25.92 c	17.42	817.32 bc
	II	69.05 ab	14.16	36.00 a	28.19 b	10.42	1201.72 ab
	III	58.00 bc	11.35	28.90 b	29.04 b	22.43	975.52 bc
	IV	47.45 c	12.21	29.74 b	26.18 c	7.93	1580.41 a
	V	73.58 a	12.73	28.41 bc	28.44 b	20.61	554.90 c
	VI	49.34 c	12.03	23.54 d	22.54 d	18.78	1034.74 bc
	VII	70.03 a	13.00	29.25 b	30.88 a	14.74	654.68 c
**Organic**	I	72.89 ab	14.77	31.26	28.48 ab	22.02 a	265.01
	II	62.59 c	14.06	35.18	25.5 bc	8.00 b	303.05
	III	60.66 c	11.81	27.83	26.46 bc	4.89 b	1002.36
	IV	64.83 bc	12.40	30.81	25.58 bc	12.54 b	443.21
	V	62.02 c	12.72	28.21	23.32 c	11.04 b	170.97
	VI	66.55 abc	12.80	26.30	27.57 ab	11.00 b	207.77
	VII	73.76 a	12.38	29.92	31.18 a	12.43 b	290.88

Letters indicate different groups at the *p* ≤ 0.05 level. I: Sürmeli, II: Tatlı Sivri Çetinel, III: İnce Sivri Kıl Tatlı, IV: Burdem 016 Demre, V: Yalova Çorbacı Sarı Sivri, VI: Demok, VII: Burkalem.

## Data Availability

Data are contained within the article.

## References

[1] Kumar A., Chandel N. (2024). Organic farming vs. integrated nutrient management: A comparative review of agricultural productivity and sustainability. Int. J. Plant Soil Sci..

[2] Singh Y.K., Rakesh S., Singh B. (2024). Organic farming for residue-free production. Int. J. Exp. Agric. Int..

[3] Kapoor N., Kumar P. (2018). Organic farming, sustainability and health. COJ Rev. Res..

[4] Willer H., Yussefi M., Sahota A., Huber B. (2023). The World of Organic Agriculture: Statistics and Emerging Trends.

[5] Tıraşcı S., Erdoğan Ü., Aksakal V. (2020). Türkiye’de organik tarım. Türk Tarım-Gıda Bilim Teknol. Derg..

[6] Akyüz N., Theuvsen L. (2021). Organic agriculture in Turkey: Status, achievements, and shortcomings. Sustainability.

[7] Türkiye İstatistik Kurumu (TÜİK) (2026). Bitkisel Üretim İstatistikleri.

[8] Duman İ. (2009). Organik biber (*Capsicum annuum* L.) tohumu üretiminde verim ve kalite özelliklerinin belirlenmesi. Ege Üniv Ziraat Fak. Derg..

[9] García-García J., García-García B., Gázquez J., Camacho-Ferre F. (2022). Sustainability assessment of greenhouse pepper production scenarios in Southeastern Spain. Agronomy.

[10] Fratianni F., Cozzolino A., d’Acierno A., Nazzaro F., Riccardi R., Spigno P., Pane C. (2020). Biochemical characterization of traditional varieties of sweet pepper (*Capsicum annuum* L.). Antioxidants.

[11] Hamed M., Ayadi M., Ben Mansour R., Gargouri M. (2019). Capsaicinoids, polyphenols and antioxidant activities of *Capsicum annuum*: Effect of ripening stage and cooking. Antioxidants.

[12] Yıldız M., Aydemir T., Ateş S., Çiftçi V., Aydın Y. (2015). Genetic diversity in Turkish pepper germplasm using iPBS retrotransposon markers. Biochem Syst. Ecol..

[13] Pereira-Dias L., Vilanova S., Fita A., Prohens J., Rodríguez-Burruezo A. (2019). Genetic diversity in a collection of pepper (*Capsicum* spp.) landraces from Spain. Hortic. Res..

[14] Dilrukshi W.M.D., Dahanayake N., Balasooriya A.S., Priyadarshana U. (2024). Analyze the seed quality of *Capsicum annum* (Clv, GROSSUM) by maintaining an optimum number of fruits load in the plant. Trop. Agric. Res. Ext..

[15] Duman İ., Aksoy U., Altındişli A., Elmacı Ö. (2018). A long-term trial to determine variations in the yield and quality of a processing type pepper (*Capsicum annuum* L. cv. Yalova yağlık-28) in organic and conventional farming systems. Agric.

[16] Julien D., Rahman M., Adebayo A., Tariq M. (2019). Organic and inorganic nutrient sources influenced growth, flowering, and yield parameters in pepper cultivars. Int. J. Agron. Agric. Res..

[17] Dzib-Ek M.G., Andueza-Noh R.H., Garruña R., Zavala-León M.J., Villanueva-Couoh E., Rivera-Hernández B., Torres-Cab W.J., Alvaro-López C.J., Ruíz-Santiago R.R. (2025). Influence of fruit ripeness on physiological seed quality of maax pepper (*Capsicum annuum* L. var. glabriusculum). Agronomy.

[18] Lammerts van Bueren E., Osman A. (2002). Organic plant breeding and propagation: Concepts and strategies. NJAS Wagening J. Life Sci..

[19] Crowder D., Reganold W. (2015). Financial competitiveness of organic agriculture on a global scale. Proc. Natl. Acad. Sci. USA.

[20] Demirkaya M., Güneş A. (2022). Effects of different effective microorganism and nitrogen fertilization doses on seed yield and quality of pepper (*Capsicum annuum* L.) plants. Gesunde Pflanz..

[21] MGM (2024). İklim Verileri.

[22] ISTA (2023). International Rules for Seed Testing.

[23] Kaya G. (2019). The use of germination indices for salinity tolerance classification of pepper cultivars. Turk. J. Agric. Food Sci. Technol..

[24] Ellis R., Roberts H., Hebblethwaite P.D. (1980). Towards a rational basis for seed testing in seed production. Seed Production.

[25] Kacar B., İnal A. (2010). Plant Analysis.

[26] Sokal R.R., Rohlf F.J. (2012). Biometry: The Principles and Practice of Statistics in Biological Research.

[27] Montgomery D.C. (2019). Design and Analysis of Experiments.

[28] Duncan D.B. (1955). Multiple range and multiple F tests. Biometrics.

[29] Bicikliski O., Trajkova F., Mihajlov L. (2018). Morphological characteristics of some pepper genotypes (*Capsicum annuum* L.) grown in conventional and organic agricultural systems: Comparative analysis. Ann. Res. Rev. Biol..

[30] Rokayya S., Khojah E. (2016). Physical-mechanical estimation of pepper (*Capsicum annuum* L.) fruit varieties. J. Northeast Agric. Univ..

[31] Sasu L., Madoșă E., Velicevici G., Ciulca S., Avădanei C., Gorinoiu G. (2013). Studies regarding correlations between the main morphological traits in a collection of bell pepper (*Capsicum annuum* var. *grossum*) local landraces. J. Hortic. For. Biotechnol..

[32] Luitel B., TaekJong L., WonHee K. (2011). Variation for fruit yield and quality characteristics in sweet pepper (*Capsicum annuum* L.) germplasm collection. Korean J. Breed..

[33] Rafiq M., Guo M., Shoaib A., Yang J., Fan S., Xiao H., Chen K., Xie Z., Cheng C. (2025). Unraveling the hormonal and molecular mechanisms shaping fruit morphology in plants. Plants.

[34] Zhigila D., AbdulRahaman A., Kolawole O., Oladele F. (2014). Fruit morphology as taxonomic features in five varieties of *Capsicum annuum* L. Solanaceae. J. Bot..

[35] Ayala-Villegas M.J., Ayala-Garay Ó.J., Aguilar-Rincón V., Corona-Torres H. (2014). Seed quality evolution of *Capsicum annuum* L. through different fruit development stages. Rev. Fitotec. Mex..

[36] Tetteh R., Aboagye L., Darko R., Osafo E. (2019). Physiological seed quality in relation to maturity stage in two pepper (*Capsicum annuum* L.) cultivars. Indian J. Agric. Res..

[37] Arslan Z., Yılmaz M., Demir İ. (2021). Farklı biber (*Capsicum annuum* L.) genotiplerinin morfolojik ve tohum verimi yönünden karşılaştırmalı analizi. J. Agric. Sci..

[38] Chakrabarty S., Islam A. (2017). Selection criteria for improving yield in chili (*Capsicum annuum*). Adv. Agric..

[39] Kaya S., Demir İ. (2020). Patlıcanda (*Solanum melongena* L.) farklı genotiplerin morfolojik ve tohum özelliklerinin incelenmesi. J. Hortic. Sci..

[40] Başay S. (2020). Organik tarımda sürdürülebilirlik için biberde (*Capsicum annuum* L. var. Kandil Dolma) organik tohum üretiminin değerlendirilmesi. Alatarım.

[41] Martínez J.S.J., Aquino-Bolaños T., Ortiz-Hernández Y.D., Cruz-Izquierdo S. (2019). Características de fruto y semilla de chile huacle (*Capsicum annuum* L.) producido en hidroponia. Idesia.

[42] Yildirim K., Demir I. (2020). The effect of maturation stage and after-ripening on seed quality in organically-and conventionally-produced pepper (*Capsicum annuum* L.) seeds. Plant Breed. Seed Sci..

[43] Nogueira J.L., da Silva B.A., Mógor Á.F., Grzybowski C.R.d.S., Panobianco M. (2017). Quality of organically produced bell pepper seeds. J. Seed Sci..

[44] Mahmud N.U., Chakma R., Ahmed N.U., Zaman M.A.U., Hossain A. (2017). Effect of sowing date on quality seed production of sweet pepper in Bangladesh. Prog. Agric..

[45] Tesi R., Lenzi A., Lombardi P. (2014). Influence of greenhouse climate on nutrient uptake in pepper (*Capsicum annuum* L.). J. Plant Nutr..

[46] Taiz L., Zeiger E., Møller I., Murphy M. (2015). Plant Physiology and Development.

[47] Drinkwater L., Wagoner P., Sarrantonio M. (1998). Legume-based cropping systems have reduced carbon and nitrogen losses. Nature.

[48] Marschner P. (2012). Marschner’s Mineral Nutrition of Higher Plants.

[49] Flores P., Carvajal M., Cerdá A., Martínez V. (2004). Nutrient uptake and yield of pepper plants as affected by salinity. J. Plant Nutr..

[50] Rubio J., García-Sánchez F., Rubio F., Martínez V. (2010). Yield, blossom-end rot incidence, and fruit quality in pepper plants under moderate salinity are affected by K^+^ and Ca^2+^ fertilization. Sci. Hortic..

[51] Eifediyi E.K., Ilori G.A., Ahamefule H.E., Imam A.Y. (2021). The effects of zinc biofortification of seeds and NPK fertilizer application on the growth and yield of sesame (*Sesamum indicum* L.). Acta Agric. Slov..

[52] Ihsanullah D.M., Magsi M.A., Shekh Z.A., Kaleri A.A., Awan M.H., Kumar V., Baloch N., Kaleri M.K., Ali A., Ali A. (2023). Growth and yield response of sesame to different nitrogen levels. J. Arable Crops Mark..

[53] Ashenaf M., Debasu T., Alemu D., Lemma E., Belete A., Awgchew H. (2025). Evaluation of nutrient role on quality, seed produce, and yield constituents of linseed (*Linum usitatissimum* L.) through nutrient omission, Central Highland of Ethiopia. Int. J. Agron..

[54] White P., Brown P. (2010). Plant nutrition for sustainable development and global health. Ann. Bot..

[55] Smith S., Read E. (2008). Mycorrhizal Symbiosis.

[56] Richardson A.E., Barea J., McNeill M., Prigent-Combaret A.M. (2009). Acquisition of phosphorus and nitrogen in the rhizosphere and plant growth promotion by microorganisms. Plant Soil.

[57] Wang D., Li P., Zhou Y. (2018). Potassium nutrition and its role in plant stress tolerance. Front. Plant Sci..

[58] Grattan S., Grieve C. (1999). Salinity–mineral nutrient relations in horticultural crops. Sci. Hortic..

[59] Reganold J., Wachter J. (2016). Organic agriculture in the twenty-first century. Nat. Plants.

[60] Alloway B. (2013). Heavy Metals in Soils: Trace Metals and Metalloids in Soils and Their Bioavailability.

[61] Kabata-Pendias A. (2011). Trace Elements in Soils and Plants.

[62] López-Millán A.F., Grusak M.A., Abadía A., Abadía J. (2016). Iron deficiency in plants: An insight from proteomic approaches. Front. Plant Sci..

[63] White P., Broadley J. (2011). Physiological limits to zinc biofortification of edible crops. Front. Plant Sci..

[64] Mengel K., Kirkby E. (2001). Principles of Plant Nutrition.

